# ‘Play the fragrance’: Designing musical soundscapes to match fragrances based on olfactory-auditory crossmodal correspondences

**DOI:** 10.1177/20416695251409265

**Published:** 2026-01-29

**Authors:** Charles Spence, Nicola Di Stefano, Felipe Reinoso-Carvalho, Bruno Mesz, Asterios Zacharakis

**Affiliations:** 1 Department of Experimental Psychology, Crossmodal Research Laboratory, Oxford University, Oxford, UK; 2 Institute of Cognitive Sciences and Technologies, National Research Council, Rome, Italy; 3 2799 School of Management, Universidad de los Andes, Bogotá, Colombia; 4 Universidad Nacional de Tres de Febrero, 28242 Instituto de Arte y Cultura (IIAC), Buenos Aires, Argentina; 5 School of Music Studies, Aristotle University of Thessaloniki, Thessaloniki, Greece

**Keywords:** crossmodal correspondences, perfume, music, emotional mediation, sensory brightness, intensity, timbre

## Abstract

In recent years, numerous studies demonstrating the crossmodal correspondences between individual olfactory stimuli and both auditory and visual stimuli have been published. However, most commercial perfumes are more complex (both chemically and perceptually) than individual olfactory stimuli, incorporating designated top, middle, and base notes. What is more, it is unlikely that it will be possible to discriminate effectively at a population level between hedonic responses to, and rated intensity of, most commercial perfumes (given that they are deliberately created to be pleasant and to provide an intense and long-lasting scent). Perfumes, unlike other classes of olfactory stimuli, also tend to be strongly gendered (masculine, feminine, or occasionally unisex). As such, the matching of music to fine fragrance faces different challenges than when matching music to the aromas and flavours of food and drink (a much more common application domain for crossmodal correspondences research currently). In this review, we examine the emerging literature on crossmodal correspondences to assess whether empirical findings can provide any actionable insights when it comes to assisting those wishing to design music and soundscapes that, in any meaningful sense, translate a perfume into its auditory equivalent.

## How to Cite this Article

Spence, C., Di Stefano, N., Reinoso-Carvalho, F., Mesz, B., & Zacharakis, A. (2026). ‘Play the fragrance’: Designing musical soundscapes to match fragrances based on olfactory-auditory crossmodal correspondences. *i-Perception*, 17(1), 1–33. https://doi.org/10.1177/20416695251409265

## Introduction

Practitioners and artists have long been interested in creating connections between olfactory and auditory domains, namely between fragrances and musical notes (see Piesse, 1867, for one of the earliest examples). Importantly, however, these historic, often intuitive and likely idiosyncratic approaches have recently been revived in the scientific literature with the emergence of research documenting systematic crossmodal correspondences – that is, associations between features across different modalities (vision, sound, taste, touch, and smell) – whose (in)congruent use may lead to altered consumer perception and attitudes, amongst other effects (Spence, 2011, 2020a, 2020b, 2020c). This interest has naturally developed into an emerging fascination concerning the putative nature of any crossmodal correspondences that can be identified between fragrance and sound (or musical features, such as pitch, timbre, etc.), and/or between perfume (as a deliberately selected combination of olfactory notes) and deliberately composed instrumental music (e.g., Di Stefano et al., 2022; Mesz et al., 2023; Velasco & Spence, 2024; and see Mesz, 2024, for a historical/anthropological perspective of the interrelations between music and scent, with a focus on crossmodal correspondences in classical and contemporary music). Indeed, the last few years have seen a growing interest in the selection, or creation, of music/soundscapes to match the profile of a given fragrance. While traditionally this may have been achieved on the basis of nothing more than artistic intuition, the development of creative compositions that are based on (or inspired by) the scientific matching of sensory features associated with fragrance and sound, and/or with the emotional feelings that they may trigger has gained significant traction in the academic literature recently (e.g., Di Stefano, 2023; Spence, 2021; Velasco & Spence, 2024).

As an example of this emerging area of crossmodal artistic endeavour, consider only the fragrance Spicebomb Infrared by Viktor & Rolf (in collaboration with L’Oréal Luxe Division), that featured an original soundtrack created by IRCAM Amplify. (Listen to the sonic logo at: https://iff.showpad.com/share/F6HYrnllRohjUvqJGYNVE.) According to the press coverage accompanying this commercial activation, sounds were selected to convey the perfume's hot and spicy notes (L’Oréal Group, 2021). Edelson (2021) writes of how: ‘The sound IRCAM Amplify sound designer Romain Barthélémy created with IFF's^1^ Christophe Hérault “is a combination of all the sounds that bring the same kind of atmosphere. It's a mix of creative sound and ambient sound, like you’d hear in a nightclub or on the street. We mix all that and create the specific sound for the specific scent.” … The beauty of what it allows brands to do in the future is establish the universal correlation between the world of scents and the world of sounds… We believe it constitutes a real revolution for e-commerce and perfume sampling. Working with a sound that generates the same sensation as the original scent means you can eliminate the hurdle of not being able to literally smell the scent because you can now hear the fragrance’. Whether such marketing claims, or the underlying approach to crossmodal matching, would necessarily stand up to the rigorous peer-review process remains to be seen.

IFF entered into a partnership with IRCAM Amplify to explore the emotional connections between sound and scent. They reported a study showing that a purposefully designed sound increased scent purchase intent by nearly 60 percent in an e-commerce site. Such results align with the following intuitive claim from Jean-Christophe Hérault, an IFF master perfumer who worked on Spicebomb with perfumer Carlos Benaim: ‘There are a lot of bridges between perfumery and music’ (as quoted in Edelson, 2021). That said, similar claims have been often considered as problematic in the academic context, where sensory translation turns out to be a much more challenging endeavour than many of those who work in the marketing/commercial field have necessarily realized (see Spence & Di Stefano, 2024a).

In 2008, the Royal College of Music in London held The Sound of Perfume competition. The challenge for the competitors was to create a composition to match the scent of Clive Christian No. 1 (see Spence, 2021). These two complementary examples, one commercial, and the other more academic, illustrate the growing interest across various sectors in fragrance-music matching.

In fact, over the years, there have been several attempts to create, or literary mentions of, scent organs (Goeltzenleuchter, 2017: Lee, 2013; Mesz, 2024; Mesz & Tedesco, 2025; see also Krause, 2022), as most famously described by Aldous Huxley in his Brave New World (Huxley, 1932; see also Huysmans, 1884/1926). However, as Avery Gilbert (2008, p. 150) notes when discussing Huxley's imagined scent organ: ‘Even if the scent organ delivered odours with the brisk precision that Huxley imagined, the audience would have trouble keeping up… The human nose works on a longer time scale; it can’t follow a *smellody* the way the ear follows a tune. Anything faster than a *largo ma non troppo* would leave an audience in the dust’. And, with more than 3,500 perfumes launched within the first decade of the twenty-first Century alone (see Zarzo & Stanton, 2009), it is obviously going to be a very challenging exercise to try to match more than a small number of fragrances with music (though, at the same time, perhaps a tremendous opportunity for crossmodal research).

### Play That Fragrance: Systematically Matching Music to Fragrance

There are a number of challenges as well as opportunities when it comes to the matching, or composition, of music to align with a specific fragrance (see Spence et al., 2024, for a recent review). On the one hand, there is likely to be less opportunity to differentiate the stimuli based on their pleasantness, or in terms of any marked differences in stimulus intensity (cf. Persson, 2011; though see Spence & Di Stefano, 2024a; 2024b), as these are likely to be closely aligned when fragrances are selected from the commercial fragrance category (Edwards, 2008; Zarzo, 2008). And where pleasantness ratings do vary, that is likely to be subjective and hence difficult to capture at a group level. Conversely, a commercial fragrance is unlikely to be experienced in isolation (e.g., in a commercial setting, as when a consumer is trying to decide which fragrance to buy, say). That is, most fragrances presented to market typically already have a back-story, a characteristic visual appearance, and may have been composed to evoke a certain emotion, mood, or setting. Consider here only how in laboratory research on crossmodal correspondences with specific olfactory notes, care is normally taken to hide the visual appearance (and any branding or descriptive information) from the participant, for fear that any crossmodal match may be based on visual appearance (e.g., colour) rather than on the purely sensory aspects of the fragrance itself, given that we are all visually dominant (Hutmacher, 2019; Spence, 2020b).

Relevant here, even the sound of the name given to a fragrance can sometimes be used to set expectations regarding what a product will smell like (Carnevale et al., 2023). Furthermore, gender is associated far more prominently with commercial fragrances than with many other classes of olfactory stimuli. Commercial fragrances are very often associated with brands that may have a strong brand identity too, and hence consumers might be primarily motivated to choose a particular (fragrance) brand rather than necessarily focusing solely on the sensory qualities of the perfume itself. What this means, in practice, is that non-sensory factors may play a more important role in determining what consumers perceive to be a good crossmodal match that is the case for other kinds of olfactory stimuli.

### Perfume-Music Matching: Challenges Highlighted Along the Consumer Journey

Before moving on to examine the empirical results and conceptual challenges of the literature on olfactory-auditory correspondences, it is worth briefly taking a closer look at the typical consumer journey when a perfume is purchased. Doing so will provide a practical exemplification of several of the conceptual issues/problems associated with matching music to a commercial fragrance that have been raised so far. Given the multiple levels at which consumers may end-up establishing correspondences with common perfumes (both sensory and semantic), it is important to think about the sequence in which they are likely to experience the various cues (including those that are non-olfactory, and indeed those that are non-sensory), as this may ultimately determine the basis on which any crossmodal match with the perfume is established (see Langner, 1997, for an early attempt to match fragrances to abstract patterns/visual displays; see also Adams & Doucé, 2017; Deroy et al., 2013; Levitan et al., 2015; Murari et al., 2014; Murari et al., 2015; Rit et al., 2019; Rodríguez et al., 2023; Ward et al., 2021a).

If one thinks about the typical customer journey as an example (e.g., Mann, 2024), the consumer would nowadays likely see the product advertised online first (presumably accompanied by the digital copy associated with each fragrance). Next, during consideration/evaluation/decision-making they may perhaps listen to the music/soundscape that has been created to evoke a specific fragrance (Mahdavi et al., 2020). Finally, they could decide to purchase the fragrance associated with a specific soundscape. In this case, they might eventually stop to consider how good a match the music/soundscape is to their idea of what the fragrance was going to smell like (based on the online copy seen on the website). Hence, while it may be possible to match music to fragrance on the basis of crossmodal correspondences that have been established at the level of individual sensory elements of the component olfactory and auditory stimuli, it is also important to recognize that this is but one part of the crossmodal communication that is both possible and likely, especially when working with an actual fragrance product (i.e., where semantic meaning and associations may also play an important role).

### Sonic Seasoning

Finally, it is worth briefly mentioning the literature on ‘sonic seasoning’. This is the name given to a subset of the research literature on crossmodal correspondences where researchers attempt to modify the perceived taste and flavour of food and drink through the simultaneous presentation of music/soundscapes. Given that 75–95% of what we think we taste comes from the aroma of food and drink (see Spence, 2015, for a review of this claim), this body of research will likely contain useful insights when it comes to matching music to fragrance, especially, perhaps, in the case of so-called gourmand fragrances (Bunschoten, 2024). The suggestion is that elements of the music may draw a taster's attention implicitly to some element of their tasting experience and/or modulate the hedonic response (though see also Wang et al., 2020). Currently, there are many examples demonstrating that people consensually match music and other sonic snippets to basic tastes, as well as to temperature cues (Murari et al., 2014; Wang & Spence, 2017c), spiciness (Wang et al., 2017), and even creaminess (Reinoso Carvalho et al., 2017). Much of the emerging research that has been published to date shows that by playing matching vs. mismatching music, it is possible to modify a consumer's tasting experience by as much as 10–15%, with such effects having been documented in both the sensory discriminative and hedonic domains (e.g., Spence et al., 2013).

The research further suggests that the matching of sonic qualities for those aromas that are commonly associated with a basic taste can typically be approximated to the musical parameters that have been established for the latter. So, for example, the musical match for a vanilla aroma/flavour can be approximated to the musical match for a sweet taste, given the two so often co-occur together in food (Spence, 2022a), while the citrus aroma can be approximated to the music that matches acidity or sourness (see Bronner et al., 2012). While one might be tempted to think of this as a kind of crossmodal matching based on transitivity, it could equally well reflect the perceptual similarity that is uniquely found in those odours that are commonly paired with a specific perceptible taste (see Spence, 2022b).

## Crossmodal Matching of Perfumes to Musical Features

There are therefore several sources of information concerning commercial fragrances that can potentially be used to feed into the recommendation process when attempting to specify a musical match: 1) Whether the fragrance is masculine or feminine (or at least is advertised as such); 2) The colour (palette) that happens to be associated with a perfume (and/or its branding); 3) Any emotional associations with fragrances upon sniffing them and/or the emotional association of the back-story that may have been created for the perfume brand; and 4) The specific olfactory notes (ingredients or essential oils that form the olfactory pyramid – a classification of olfactory notes that make up a perfume) that happens to be associated with each fragrance. The first three of these will be dealt with in the following Sections (Matching Music for Masculine/Feminine Scents, and Musically Matching Emotional Associations with Fragrances), while the fourth is addressed in the Section Creating a Musical Menu Corresponding to the Olfactory Notes in a Fragrance when looking at the evidence concerning cross-sensory matching. Of course, there may not always be specific (or unique) musical recommendations for each of the sources of information identified concerning potential correspondences with specific fragrance materials.

### Matching Music for Masculine/Feminine Scents

Perfume's positioning as masculine or feminine (or gender neutral) will likely affect the customers’ semantic associations with the scent (e.g., Iseki et al., 2021; Krishna et al., 2010). According to the results of one study, the faster the tempo, the more sensitive/dramatic, and the more assertive/masterful the music, the more masculine it is rated (Sergeant & Himonides, 2016). By contrast, the more sensitive/emotional the music, the more feminine it is judged as being. Elsewhere, the level of perceived femininity/masculinity of a fragrance has also been found to be correlated with the organization of auditory pitch. In particular, in a study in which 14 pianists (2 female) improvised music that they associated with a collection of scents used in perfumery, feminine odours were characterized by a musical match that was negatively correlated with the ambitus of the improvisation (defined as the difference between the highest and lowest note), while being positively correlated with pitch-class entropy, a measure related to complexity and surprise (Mesz et al., 2023). In fact, according to Taylor (2012), music can be considered as ‘a dynamic mode of gender’ and ‘an essentially gendered discourse’. What determines the gender or sexuality communicated by the sounds of music? One challenge here is that the codes in music that signify ‘masculinity’ and ‘femininity’ may change over time because the meaning of femininity/masculinity itself can also change across time and culture (Rycenga, 1994).

Researchers interested in the gender associations that the consumer intuitively assigns to a particular fragrance may want to present the fragrance blind and ask for a response on a 9-point Likert scale for gender perception of scent (‘1: extremely masculine’ to ‘9: extremely feminine’) as used in the study by Iseki et al. (2021). These scores could then be used by the sound designer to match the degree of masculinity/femininity of the music. There might also be a link to sonic matches that are associated with the concepts of heavy vs. light (Murari et al., 2014). At the same time, however, some commentators have suggested that the notion of gendered perfume may become obsolete (Grech, 2025), while the perceived gendering of music may depend on socially agreed gendered behavioural styles, whose differences are narrowing over time (Sergeant & Himonides, 2016). Therefore, gender in this crossmodal matching process should eventually benefit from further information about the target-specific group of potential consumers of the fragrance at stake (think of more advanced psycho-demographics, as well as certain criteria related to consumer attitudes, aspirations, archetypes, etc.).

### Does the Colour Associated with a Perfume Brand Influence Crossmodal Music Matching?

Colour cues have been shown to both facilitate and influence olfactory identification (e.g., Gilbert et al., 1996; Kemp & Gilbert, 1997; Levitan et al., 2014; Spence, 2020b, 2020c). Consequently, any colour cues that are linked to a given perfume and/or its branding can presumably be mapped onto different pitches, with lower pitched sounds normally associated with darker colours (e.g., Isbilen & Krumhansl, 2016; Melara, 1989; Palmer et al., 2013; Spence, 2020a). Given that the visual imagery associated with a perfume brand will likely be presented first, and given that humans are visually dominant (Hutmacher, 2019), it is important to note that a perfume brand's colour scheme may help to set a customer's expectations regarding the associated musical qualities (prior to their experience). Note that a similar point has been raised previously with regard to the matching of music to wine, namely that at least some part of the robust crossmodal matches that have been obtained may be driven by the colour of the wine (e.g., red vs. white; see Spence et al., 2013; Spence & Wang, 2015a, 2015b, 2015c; though see Wang & Spence, 2018, for evidence that specific music is associated with particular wine, even when the colour of the latter is controlled).

### Musically Matching Emotional Associations with Fragrances

Emotional, or affective, valence has often been reported to be the mediator for many crossmodal correspondences (Di Stefano et al., 2024; Helmefalk & Hultén, 2017; Palmer et al., 2013; Schifferstein & Tanudjaja, 2004; Wang & Spence, 2017b; see Spence, 2020a, for a review). One particularly fruitful approach that has been used by researchers to assess the semantic/affective connotations of a stimulus or concept involves the use of the semantic differential (SD) (Osgood et al., 1957; Snider & Osgood, 1969). This approach provides a SD analysis of the terms (multiple bipolar adjective scales) with people's ratings often being reduced down to just two or three dimensions, namely Evaluation, Potency, and Activity (e.g., Park et al., 2021). Music/soundscapes could then be composed in order to match the SD profile associated with a particular perfume (cf. Kawachi et al., 2011; Miller, 2021; Oyama et al., 1998; Watt & Ash, 1998; Watt & Quinn, 2007; see also Higuchi et al., 2004; Yoshida, 1964). Yoshida used the SD technique with 30 naïve female Japanese participants who rated a range of familiar odorants. Following factor analysis, the SD technique (incorporating 25 bipolar adjective scales in this case) yielded three factors which Yoshida chose to label as: 1) sensory pleasure; 2) harshness; and 3) intensity or vividness.

Dalton et al. (2008) conducted a SD analysis of various sets of fragrances. In this study, the participants were required to rate each of ten olfactory stimuli, with 100 participants rating a separate set of 10 odorants (e.g., lemon, leafy green, peppermint, lavender, eucalyptus, patchouli, rose, muguet, peach, and banana) that were matched in terms of their relative intensity on 7-point scales, anchored by the two adjectives in each pair. Principal components analysis (PCA) was then used to reduce the responses down to a three-dimensional space with the dimensions of Activity (Low-High), Potency (Weak-Strong), and Evaluation (Positive-Negative). Only 17 of the SD adjective pairs loaded on the three dimensions that together accounted for 53% of the variance (see **Table 1**). It is interesting to see the ‘Fresh-stale’ adjective pair at the top of the first column, given its prominent role in Zarzo's (2020) contemporary classification of cosmetic fragrances. The basic idea here in terms of designing soundscapes to match fragrances is that the SD descriptors chosen for a particular fragrance could presumably also be used to match musical parameters that have similar semantic associations (Dalton et al., 2008).

**Table 1.** The 17 (Out of a Total of 50) Semantic Differential Pairs Presented to Participants in Dalton et al.'s (2008) Study Using the Semantic Differential (SD) Technique to Examine the Multidimensional Representation of Odours That Loaded on the Three-Dimensional Solution That is Common in This Space [Table Reprinted from Dalton et al., 2008)].

Factor 1 (Evaluation)	Factor 2 (Potency)	Factor 3 (Activity)
Fresh-stale	Strong-weak	Ordered-chaotic
Good-bad	Powerful-powerless	Quiet-noisy
Happy-sad	Harsh-mild	Relaxing-stimulating
Harmonious-unharmonious		Wet-dry
Healthy-unhealthy		Muddy-clear
Beautiful-ugly		
Smooth-rough		
Clean-dirty		
Safe-dangerous		

However, that said, one might question the need to use all 50 or so scales, when most SD studies show that whatever the stimulus being evaluated, the SD analysis can normally be collapsed down onto just two or three underlying dimensions (i.e., and explain some modest part of the total variance). A residual concern here, though, might be whether there is sufficient discriminability between different fragrances based on what are likely to be relatively small variations in the two or three principal dimensions that are typically revealed by the SD approach. Indeed, as we will see below, other researchers have chosen to use a reduced range of SD adjective pairs in their studies.

Another approach that is potentially worth considering here is to use odour-related emotion scales such as the Geneva Emotion and Odor Scale (GEOS, Porcherot et al., 2010) to obtain affective profiles on which to base one's music compositions. Such an approach would seem to make sense given that music-induced emotion is usually described with very similar terms. In fact, it turns out that there is a wide overlap between the terms of the GEOS and those of the GEMIAC (Geneva Music-Induced Affect Checklist, Coutinho & Scherer, 2017). Relevant here, Jacobsen et al. (2024) recently attempted to reduce the 45 initial scales of the Geneva Emotional Music Scale down to just nine items.

### Interim Summary

Taken together, the observations and research mentioned in this section highlight the multiple potential factors that might be expected to influence the design of soundscapes that correspond crossmodally with fine fragrances. The sources of information concerning commercial fragrances that may influence the musical match include whether the fragrance is masculine or feminine, the colour (palette) associated with a perfume and any emotional associations with the perfume. In the next section, we take a closer look at the specific musical qualities that are associated with specific olfactory stimuli. Indeed, recent advances in crossmodal correspondence research have attracted renewed interest in the systematic mapping between olfactory and auditory modalities, especially in the context of perfumery and music. Analogously, this framework has been extended to perfumery, where auditory features (e.g., pitch, timbre, tempo) may be matched to olfactory stimuli based on shared semantic, emotional, or structural features.

## Creating a Musical Menu Corresponding to the Olfactory Notes in a Fragrance

Crossmodal correspondences between olfactory fragrance notes and musical properties operate at multiple levels: Loudness, pitch, brightness, roughness, as well as other timbral dimensions/qualities (at least when considering the auditory modality) of individual tones, as well as several parameters referring specifically to multiple musical elements (such as the ambitus, or range of notes) and phrases, such as tempo, etc. have all been suggested (or documented) to be relevant for these correspondences over the last 150 years or so. In the sections that follow, we discuss the scientific evidence that has been published to date concerning different crossmodal matches between specific sonic and olfactory stimulus attributes, as well as highlighting the inspirational early suggestions of Septimus Piesse (1867), the English chemist and perfumer.

###  Pitch/Note–Olfactory Note Crossmodal Correspondences

**Figure 1.**
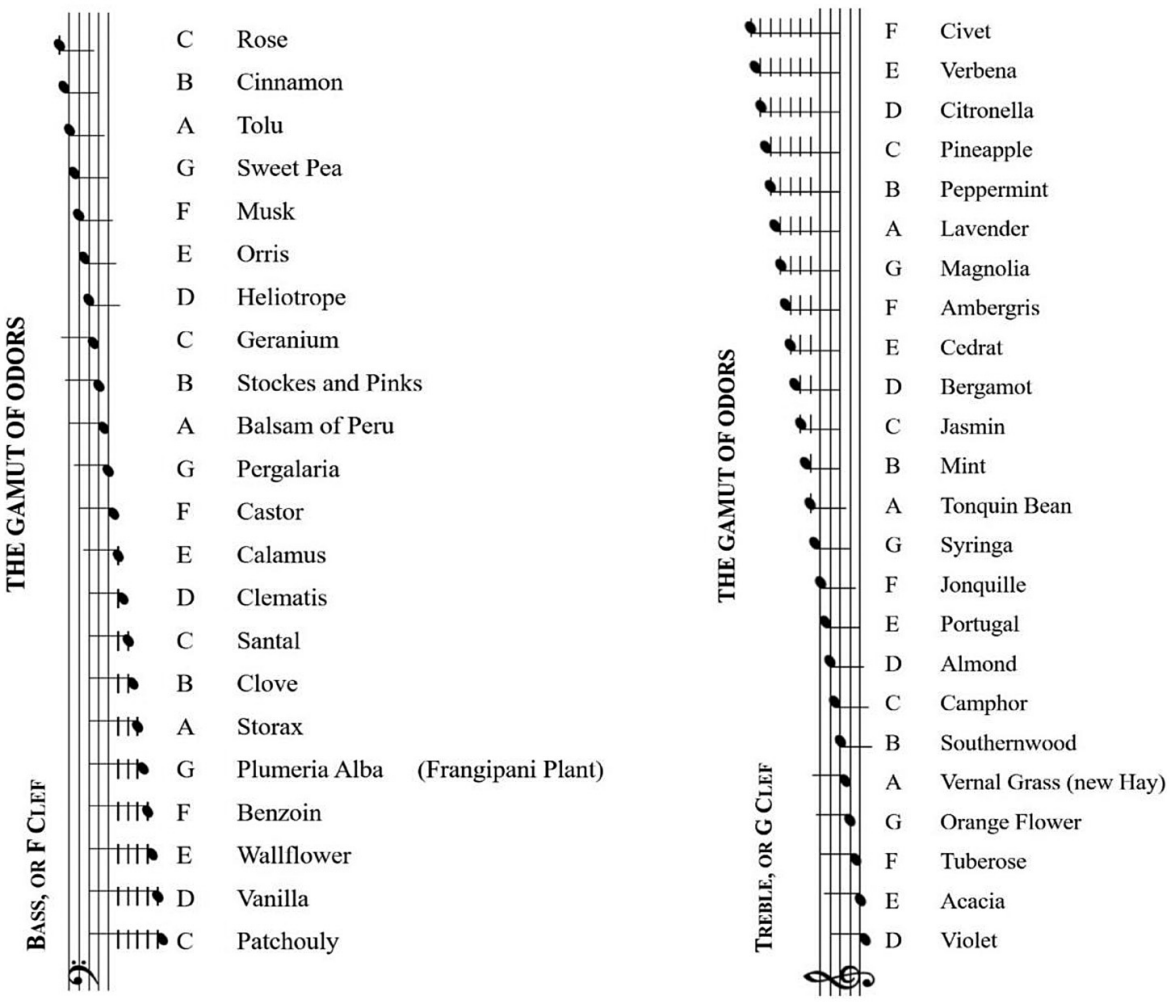
The Gamut of Odours – From Septimus Piesse (1867).

George William Septimus Piesse (May 30, 1820–October 23, 1882), known as Septimus Piesse, was a leading author and innovator of modern perfume ideas, inventing the concept of notes in perfumery that are still widely used today (see Kettler, 2015). Piesse famously proposed a crossmodal alignment of specific musical notes with specific perfumery ingredients. In particular, his ‘gamut of odours’, first appeared in print in the 1867 version of his book (see **Figure 1**). Intriguingly, the ‘Perfumery Organ, 2015/17’ displayed at the NTT Intercommunication Centre in Tokyo, Japan to recreate Piesse's Gamut of Odours, used exactly the same mapping of scents to sounds as Piesse had originally had in mind (see Spence, 2021). While Piesse was likely the first to make any concrete suggestions in this space, a closer reading of his work suggests that the chemist may actually have been more interested in the similarities between the way in which different olfactory notes combine (harmoniously) in a manner similar to the way in which musical notes combine, rather than so much on a direct crossmodal mapping of one musical note to a particular olfactory note. At this point, it is also worth noting how Piesse appears to be recommending an absolute, though likely idiosyncratic, one-to-one mapping between pitches and particular olfactory notes. Contemporary research contrasts with Piesse's suggestion, advocating that olfactory correspondences with pitch tend to be relative rather than absolute in nature (see Spence, 2019, for a review).

### Sensory Brightness

One of the earliest empirical studies on putatively ‘amodal’ perceptual dimensions (though see Spence & Di Stefano, 2024b, on the problematic notion of ‘amodal’ in the field of cognitive neuroscience) was published by Erich Von Hornbostel (1931), who believed that brightness represented ‘a universal dimension of sensory experience’. Note that for musicologists, sensory brightness is typically considered as but one component of timbre perception. However, that being said, it is worth bearing in mind that the term brightness doesn’t have quite the same connotation for perfumiers. Indeed, coffee experts (e.g., baristas) sometimes similarly talk of a coffee's ‘bright acidity’ (e.g., Nicole, 2021; https://www.sabores.co.za/2019/07/22/guide-to-colombian-coffee/#:∼:text = Flavor%20Profile,fruits%2C%20and%20hints%20of%20spice.). The Austrian musicologist had three participants match sounds of different pitches to points along a greyscale, and then match scents with greyscale values. Given the apparent transitivity between different crossmodal comparisons that Von Hornbostel's results revealed, his findings were taken to be consistent with the concept of ‘sensory brightness’ being common to all the senses, and hence a universal dimension of sensory experience. However, although the results obtained may support robust crossmodal psychophysics (between olfactory and visual brightness), it is worth noting that the crossmodal match may not be something that people are themselves subjectively confident about. As von Hornbostel (1927/1938, pp. 210-211) himself wrote almost a century ago now: ‘Anyone can find on the piano that tone which sounds as bright as lilac smells. (Generally, he thinks the task nonsense at first, but, if he can be persuaded to deal with such nonsense at all, it goes very well.)’. This statement could perhaps also be interpreted as referring to a single perceiver (i.e., in this sense meaning that ‘anyone’ can find his own tone-lilac correspondence).

Commentators were, however, quick to criticize von Hornbostel's (1931) claims concerning the existence of an absolute crossmodal mapping (Hartshorne, 1934), showing instead some relative contributions to the crossmodal matching (Cohen, 1934). Nevertheless, it is important to note that this may not undermine the general meaningfulness of the mapping, given the evidence that pitch-based crossmodal correspondences also appear relative (Spence, 2019). In fact, absolute perceptual judgments appear to be the exception rather than the norm, with some of the only examples reported in the case of synaesthetes and those individuals possessing absolute pitch (see Di Stefano & Spence, 2024).

**Figure 2.**
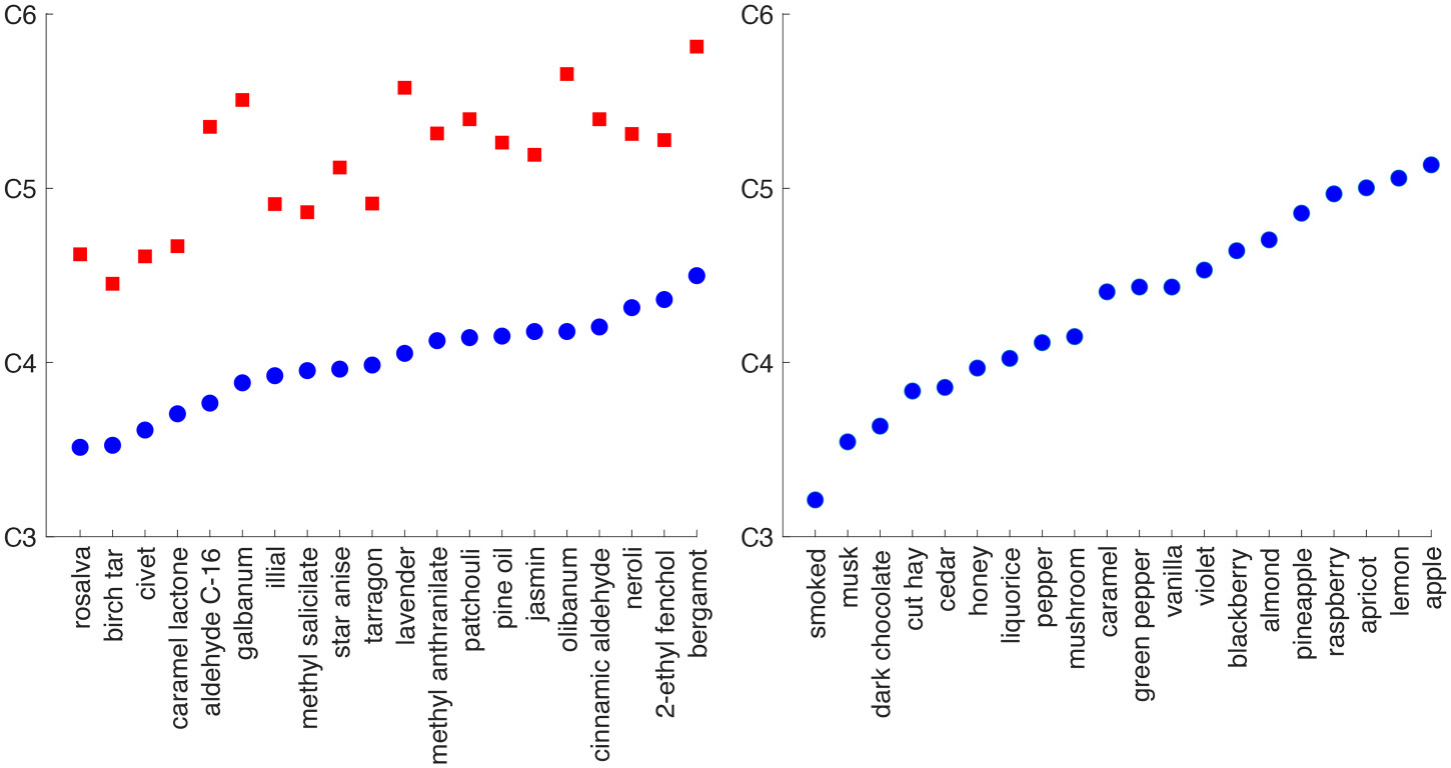
Crossmodal matchings between odours and pitch. Adapted from Belkin et al. (1997) (left) and Crisinel and Spence (2012) (right). Note that the two lines of dots in the left panel reflect the crossmodal matching results based on ascending (blue) and descending (red) staircases (i.e., starting with a low or high-pitched sound, respectively). The y-axis denotes the musical note/pitch (with a higher pitch indicated by a higher elevation on the scale. [Figure reprinted from Di Stefano et al., (2022)].

Subsequent studies have demonstrated that specific auditory pitches are indeed consistently matched to specific odorants according to their odour quality, even when odour intensity is matched, and not to their hedonic tone (Belkin et al., 1997). For instance, Crisinel and Spence (2012) conducted a study showing that British participants matched lemon and fruity scents with the highest auditory pitches, while matching the lowest pitch sounds with smoked, musk, and dark chocolate aromas instead (see **Figure 2**). These can be considered as matching the birch tar, civet, and caramel lactone in Belkin et al.'s study. Crisinel et al. (2013) also observed that those olfactory notes described as smelling of ginger, cookies, musk, and roasted coffee were significantly lower in their auditory pitch association than were those smells described as candied orange and iris flower. These findings were largely confirmed by Zacharakis (2024), who linked higher pitches to fruity and sour aromas, and lower pitches to scents such as tobacco and black pepper. Meanwhile, Mesz et al. (2023) reported a crossmodal association between higher pitches and fresher/lighter odour character, which is the most salient perceptual dimension in perfumery scents (Zarzo, 2013). This relationship is consistent with the previous studies just cited and may in fact be related to brightness by a transitivity of crossmodal correspondences, since besides odour freshness, high pitch has also been shown to be associated with visual brightness (Hubbard, 1996; Marks, 1989).

### Timbre–Olfactory Note Correspondences

Beyond intensity, auditory pitch, and sensory brightness, researchers have also established auditory–olfactory crossmodal correspondences with timbre: Musical timbre is the perceptual attribute that distinguishes sounds that are otherwise identical in pitch, loudness, duration, and spatial position. It is determined by a sound's spectral envelope (the frequency content, relative intensities, and exact frequencies of partials), temporal envelope (the evolution of overall amplitude over time), and spectro-temporal variation (the dynamic changes in spectral content over time), and more specifically with types of instrument sounds (see **Figure 3** from Crisinel & Spence, 2012; see also Crisinel & Spence, 2010; Crisinel et al., 2013).

**Figure 3.**
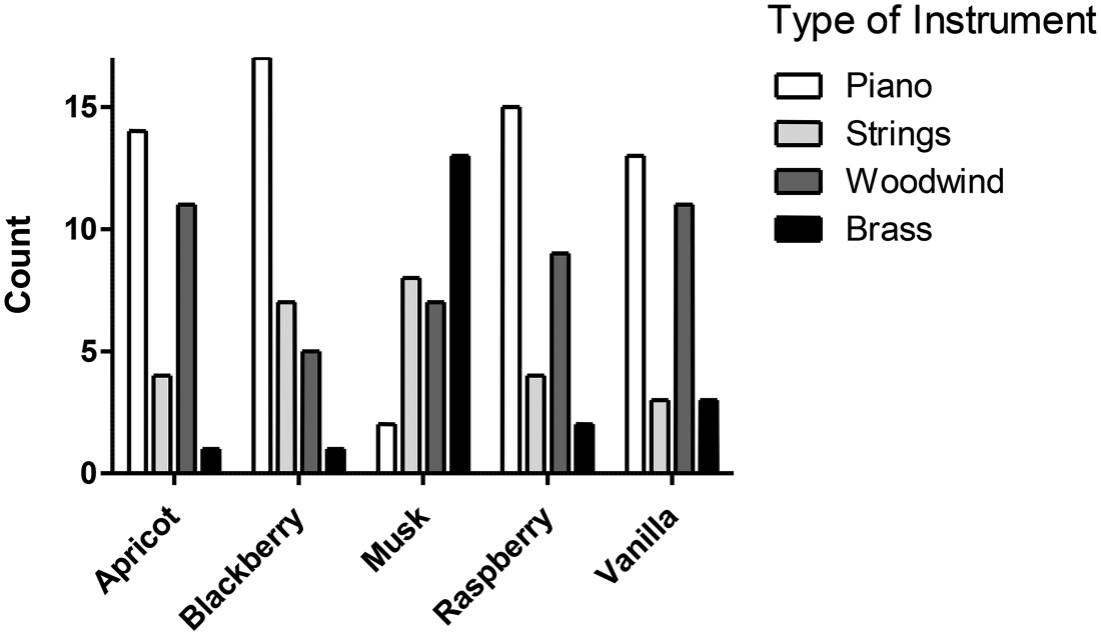
Choice of instrument as a function of the odour presented. Only odours that led to significant preferences for instruments are shown. The total count per category is 30. [Data taken from Crisinel and Spence (2012)].

In one of the first studies of its kind, Crisinel and Spence (2012) used a selection of the olfactory stimuli from *Le Nez du Vin* kit (Note, though, that the wine-related olfactory notes only partially overlap to those olfactory notes that are typically used in the world of perfumery) to provide evidence supporting the existence of a number of consensual crossmodal correspondences between instrument-class and fragrance notes. One reason that makes this initial finding noteworthy is that timbre and odour might appear as semantically unrelated. It is likely that their complexity and multidimensionality means that causes our communication to rely mainly on descriptions tied to broader or narrower source categories (e.g., ‘a fruity smell’, ‘a piano-like sound’). If one were to focus solely on such source-based semantics, it might appear that timbre–olfactory correspondences are either too weak or altogether absent. However, when a broader view of semantics is adopted – understood as the emergence of meaning through a percept evoking something other than itself (Patel, 2008) – associations based on shared abstract qualities, rather than common sources, become possible.

While such a viewpoint on timbral semantics is by no means new, tracing back to Helmholtz's (1877) pioneering work, it has recently resurfaced, becoming an increasingly intriguing area of research amongst a growing number of music scholars (e.g., Reymore, 2022; Reymore & Huron, 2020; Saitis & Weinzierl, 2019; Wallmark & Kendall, 2018; Zacharakis et al., 2014; Zacharakis et al., 2015). Music psychologists have systematized the salient semantic terms for timbre description, many of which constitute crossmodal metaphors (e.g., Wallmark, 2019). While direct commonalities between auditory and olfactory semantics are rarely reported – largely because vision and touch dominate the crossmodal mappings used to describe auditory qualities – there remain candidate qualia that may serve as a bridge between audition and olfaction. Relevant here, one might also wonder whether timbre–olfactory correspondences could be based on a more sensory mapping of roughness/astringency (see Di Stefano & Spence, 2022; Saluja et al., 2025; Sáenz-Navajas et al., 2025)?

Motivated by the conceptual problem outlined above, Zacharakis (2024) published a study in which he investigated crossmodal correspondences between timbre and scent. The 29 musically trained Greek participants who took part in this particular study listened to 26 complex synthetic tones (the sounds are available as supplementary materials to the paper itself) and had to rate their correspondence with 12 different aromatic oils comprising: Vanilla, honey, caramel, cinnamon, coffee, (black) pepper, lemon, lemon blossom, pomegranate, melon, cherry, and tobacco leaves. Analysis of the results revealed that most scents featured at least one association with a sound that was above chance. Crucially, the salient acoustical correlates of basic aromatic categories could be summarized as follows: Both fruity (e.g., cherry, melon, and pomegranate) and sour aromas exhibited a positive correlation with pitch (consistent with Crisinel & Spence, 2012; and Ward et al., 2021b). Additionally, fruity scents were more likely to be associated with sounds featuring pronounced low harmonic partials (1^st^–4^th^), low noise content, low roughness, and a greater number of distinct pitches. Conversely, sour aromas were linked with stronger energy in the higher frequencies (consistent with the earlier findings reported by Knoeferle et al., 2015; Simner et al., 2010). Sweet scents correlated with those synthesized sounds characterized by a lower spectral centroid, and positive spectral skewness (consistent with the findings reported by Bronner et al., 2012; Mesz et al., 2011). Aromas in the spicy/other category were associated with weak lower partials (fundamental frequency in particular) and stronger noisy components (noisy timbres, higher roughness, less smooth spectral envelopes, slower attacks, and longer lasting sounds; cf. Knöferle & Spence, 2012; Ward et al., 2021b). (Note also that in the context of sonic seasoning, Wang et al. (2017) established a sonic match for spicy-tasting foods).

**Figure 4.**
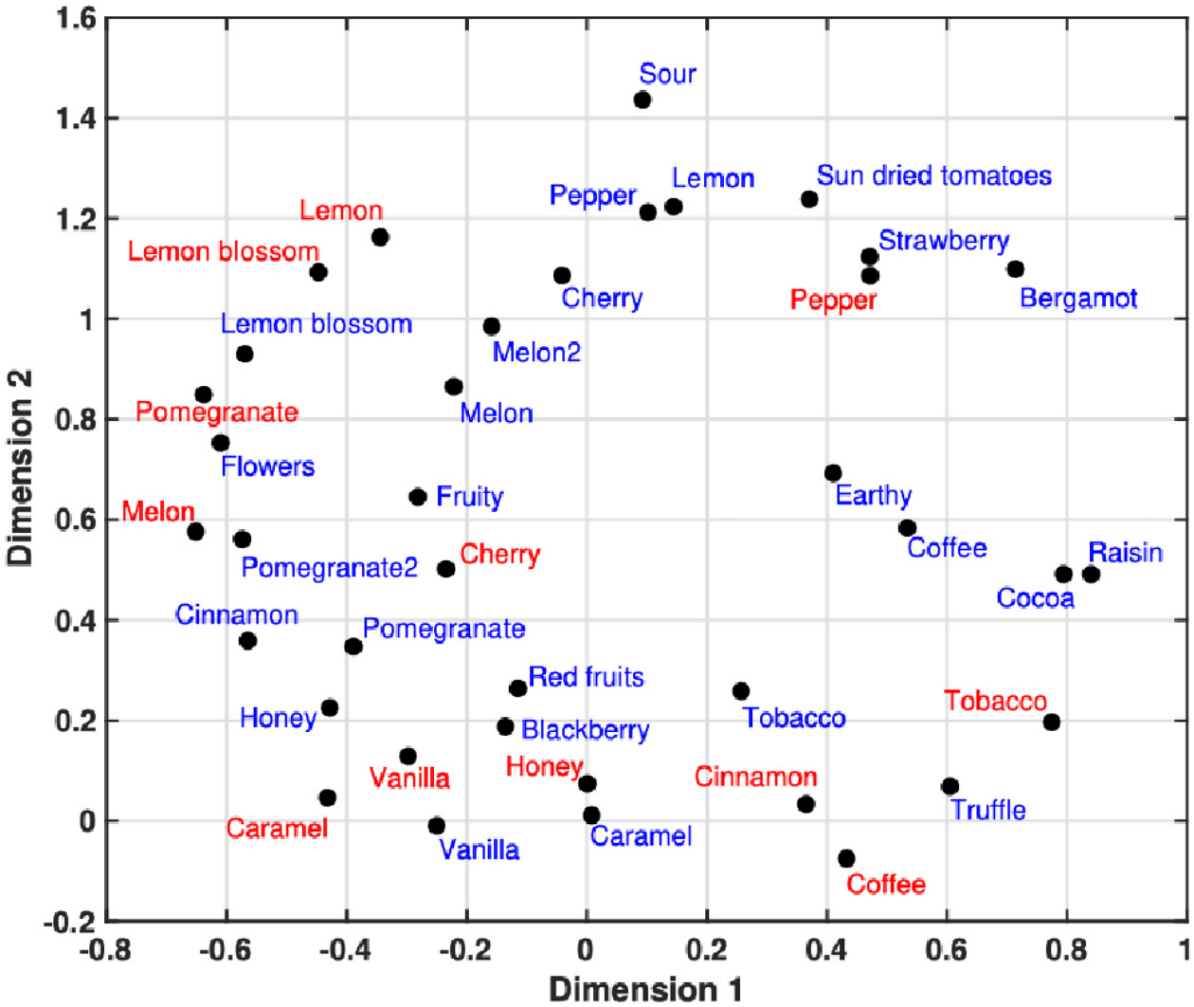
Two-dimensional semantic space derived from the conversion of semantic vectors into dissimilarities and subsequent MDS analysis. Red labels represent the aromatic stimuli and blue labels correspond to the sound stimuli, named after their intended aromatic counterparts. The double versions of sonic pomegranate and melon indicate different realizations of the same aromatic target. A tentative labelling was proposed based on the semantic profiles of individual stimuli. 1_st_ dimension: (+) complex/full vs. bright/ethereal/sweet/fresh (–); 2_nd_ dimension: (+) thin/sharp vs. warm/full/rich (–) [Data taken from Zacharakis (2025)]. MDS, multidimensional scaling.

In a follow-up study, Zacharakis (2025) went on to expand on his previous research regarding timbre-aroma crossmodal correspondences by examining the semantic mediation hypothesis, according to which crossmodal correspondences may be partly explained by the existence of common semantic qualities. In a psychophysical study, a different group of 26 musically trained Greek participants now rated the same 26 complex synthetic tones and 12 aromatic stimuli across two separate blocks of trials using a common set of semantic scales. The semantic scales were based on the inclusion of terms that could serve as common descriptors for both olfactory and auditory stimuli, namely: rich, thin, full, sweet, fresh, ethereal, woody, metallic, warm, sharp, bright, and complex. The analysis of semantic variables identified a largely consistent organization for both modalities, condensing into three prominent clusters: [*bright, fresh, ethereal*], [*sharp, metallic*], and [*full, rich, warm*]. Furthermore, the distances between stimuli that were derived from semantic ratings exhibited a strong correlation with previously estimated ground-truth distances of direct crossmodal associations. Additionally, the stimulus configuration within the semantic space generated through multidimensional scaling analysis (see **Figure 4**) exhibited notable commonalities with the organization of stimuli derived from direct timbre-aroma correspondences established by Zacharakis (2024). The first dimension of this spatial configuration was tentatively labelled complex/full vs. bright/ethereal/sweet/fresh, and the second dimension was labelled thin/sharp vs. warm/full/rich based on the individual semantic profiles of each sonic and olfactory stimulus. Taken together, therefore, the results of this study provide some compelling evidence that semantic mediation plays a significant role in shaping crossmodal correspondences between auditory and olfactory stimuli, thus paving the way for further exploration of the underlying semantic dimensions that connect these two modalities. These results likely also provide some useful insights for those wanting to design soundscapes to match specific fragrances.

### Musical Parameters–Olfactory Note Correspondences for Olfactory Freshness

After pitch and timbre, which are deemed more low-level auditory attributes, it is now time to move on to the higher level musical parameters that are linked to key scent descriptors, such as freshness. Remember here that according to Zarzo's (2008) PCA of a wide range of commercial fragrances, freshness came out as a primary dimension, followed by whether the cosmetic fragrance was masculine or feminine. Indeed, recommendations for conveying freshness in music have recently been developed. For example, Reinoso-Carvalho and colleagues conducted research to identify the sound qualities that were associated with freshness in a Brazilian commercial fragrance sample (Cantu et al., 2022; Rodríguez et al., 2024). This research presented a sonic-seasoning-based methodology for characterizing music that evoked the perception of the freshness of a fragrance. First, sound parameters that effectively triggered the concept of freshness were identified (e.g., slow tempo, woodwinds, piano, high/mid-pitch, nature sounds). Based on these results, originally composed brand-aligned songs were tested to assess associations with freshness and brand values. Finally, these songs were shown to effectively modulate the olfactory experience of two commercial fragrances. The results revealed how soundtracks can enhance the perception of freshness descriptors (e.g., sweet, fruity, cold, calm, blue, light) in the fragrance experience, thus promoting novel practical applications in multisensory strategies for the scent market (cf. Velasco-Sacristán & Fuertes-Olivera, 2006).

Taken together, the research reviewed in this section suggests that crossmodal mappings between scent and sound can operate in terms of both low-level (e.g., pitch, brightness, timbre) and higher order musical structures (e.g., tempo, instrumentation). Empirical studies demonstrate that perceptual dimensions such as freshness, sweetness, or complexity in scents align systematically with musical features such as spectral centroid, harmonic content, and temporal structure. At the same time, recent psychophysical work (e.g., Zacharakis, 2024, 2025) underscores the role of semantic convergence – shared descriptors across modalities – in organizing these associations. Ultimately, designing effective auditory–olfactory pairings for commercial or artistic applications will likely depend on integrating perceptual, affective, and semantic mappings, with particular attention being paid to any relevant cultural and contextual factors. This layered, data-informed approach will likely enable more coherent and meaningful fragrance–sound design.

## Learning from Musical Improvisations Created in Response to Scents

While several direct music–olfactory note crossmodal matches (or correspondences) have now been documented in the literature, they have largely been studied in isolation: That is, isolated olfactory notes have been paired with individual musical objects varying in terms of their intensity (loudness), brightness (pitch), and/or timbre (see Section Creating a Musical Menu Corresponding to the Olfactory Notes in a Fragrance). By contrast, commercial fragrances are typically complex olfactory compositions having multiple olfactory notes that may blend with one another and/or retain their unique identity in ways that may be hard to predict (see Barwich, 2019; Millar, 2019, on the notion of olfactory objects or Gestalts). Note that the term Gestalt refers to the perceptual grouping/segmentation of stimuli. Hence, in the present context, the notion of an olfactory Gestalt implies that the different individual notes that make up a fragrance are grouped together into some kind of unitary olfactory percept.

**Figure 5.**
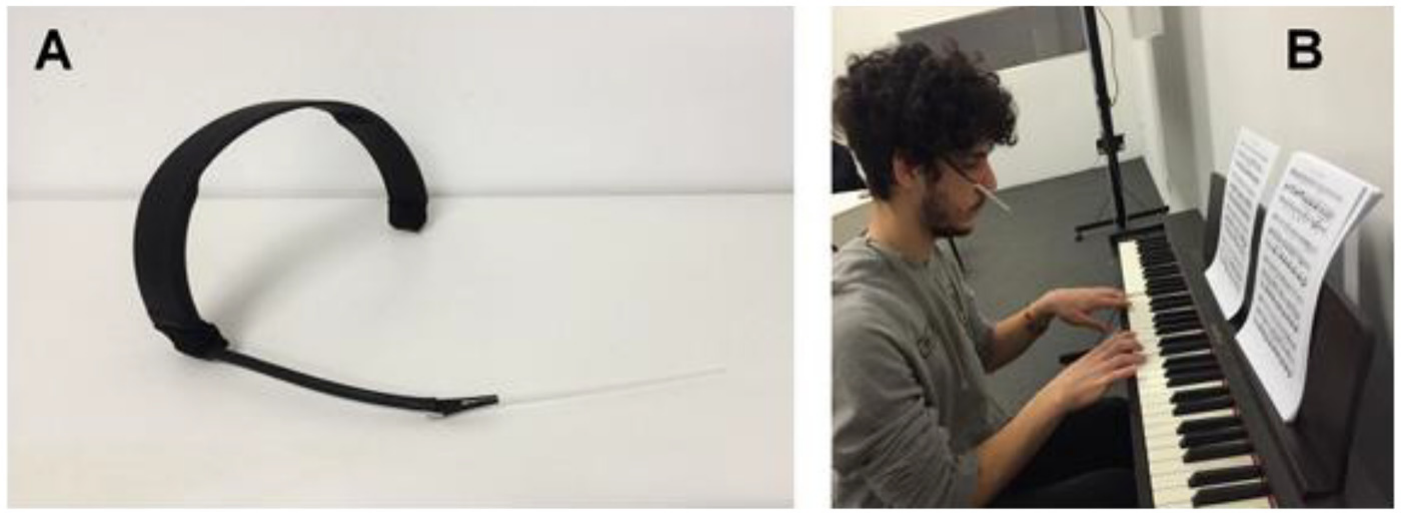
(A) Hand-made device with a clamp holding the paper strip containing the aroma. (B) Demonstration of the use of this device with participant improvising music to scent. [Reprinted from Mesz et al. (2023)].

An alternative approach to the development of musical parameters involves musical improvisation in response to (multidimensional) olfactory stimuli. This method allows researchers to capture a holistic, intuitive response to the scent as a whole, which is closer to how a consumer experiences it. So, for instance, Mesz et al. (2023) explored the crossmodal correspondences emerging from musical improvisation elicited by 20 olfactory stimuli, importantly allowing for the assessment of multiple musical parameters at once. A group of 14 pianists was asked to smell each olfactory stimulus and to play a short free improvisation inspired by it (see **Figure 5**). From each improvisation, 14 musical parameters were extracted. The same odorants were also described by a panel of 15 volunteers. The main outcomes of this study were: 1) The mean sensory ratings on a scale of fresh vs. warm appeared to be correlated with the average pitch of the improvisation; 2) The feminine odour character was negatively correlated with the ambitus of the improvisation, defined as the difference between the highest and lowest note, and was positively correlated with pitch-class entropy; 3) The four odorants that were perceived as somewhat camphoraceous, such as lavender and mint, yielded more non-legato/staccato articulation or rests in the musical improvisations; and 4) Pleasantness yielded a negative correlation with pitch-class entropy and dissonance, being positively correlated with the lowest note. The first outcome is consistent with earlier studies, while outcomes 2–4 constitute novel empirical findings. It is important to recognize how the research that has been reviewed thus far in this section only relates to the experience of smell in the moment. While these results (and the methodological approach that underpins it) are undoubtedly intriguing, it should be remembered that since all of the improvisations were executed on the piano, this approach cannot provide insights concerning fragrance-instrument type crossmodal associations (see **Table 2** for the musical correlates of scents that have been established to date).

**Table 2.** Compilation of Acoustic Attributes Linked to Olfactory Properties, Based on Current Evidence. ‘+’ Indicates a Positive Relationship, While ‘–’ Indicates a Negative one.

Acoustic Attribute											
Olfactory qualities		Pitch	Harmonic spectrum energy distribution	Roughness	Noisiness	Attack time	Melodic ambitus	Entropy of pitch classes	Staccato articulation or pauses	Dissonance	Tempo (bpm)
	Fruity	+	strong lower partials	–	–						
	Sweet		strong lower partials								
	Sour	+	strong higher partials								
	Bright	+									
	Fresh/ light	+		–							–
	Earthy/ bitter/ spicy	–	weak lower partials	+	+	+					
	Pleasant							–		–	
	Feminine						–	+			
	Masculine										+
	Camphoraceous								+		

It is interesting to consider whether memory/recollection could be relevant in determining an improvisational strategy, especially given the strong link between fragrances and memories; incidental evidence of this may be found in Mesz et al.'s (2023) study, where pianists were interviewed about the biographical/aesthetic associations that influenced their improvisations. Here, one might also consider semantic theming and the matching of scent and sound, be it by season (e.g., Christmas music paired with season-appropriate scents; see Spangenberg et al., 2005; see also Mattila & Wirtz, 2001; Morrison et al., 2011), or by country (e.g., music and scents that are commonly associated with a particular region or country; see North et al., 1997; Spence et al., 2019). More formal attempts to categorize fragrances such as Edwards’ classification (Edwards, 2008) may also inspire sound designers and composers. Pimentel et al. (2025) revealed consistent correspondences between musical modes and Edwards’ 4 fragrance families (Floral, Fresh, Oriental, Woody) and 14 subfamilies. Note that the names of most of these categories are highly suggestive of musical associations; as an example, one of the authors (BM) has composed a musical fragment intended to match clove, an Oriental smell, using Indian ragas and instruments. This music was commissioned by the European Sensory Network for a multisensory smell training program.

## What are the Fundamental Dimensions of Olfactory Experience in the Context of Fragrances?

**Figure 6.**
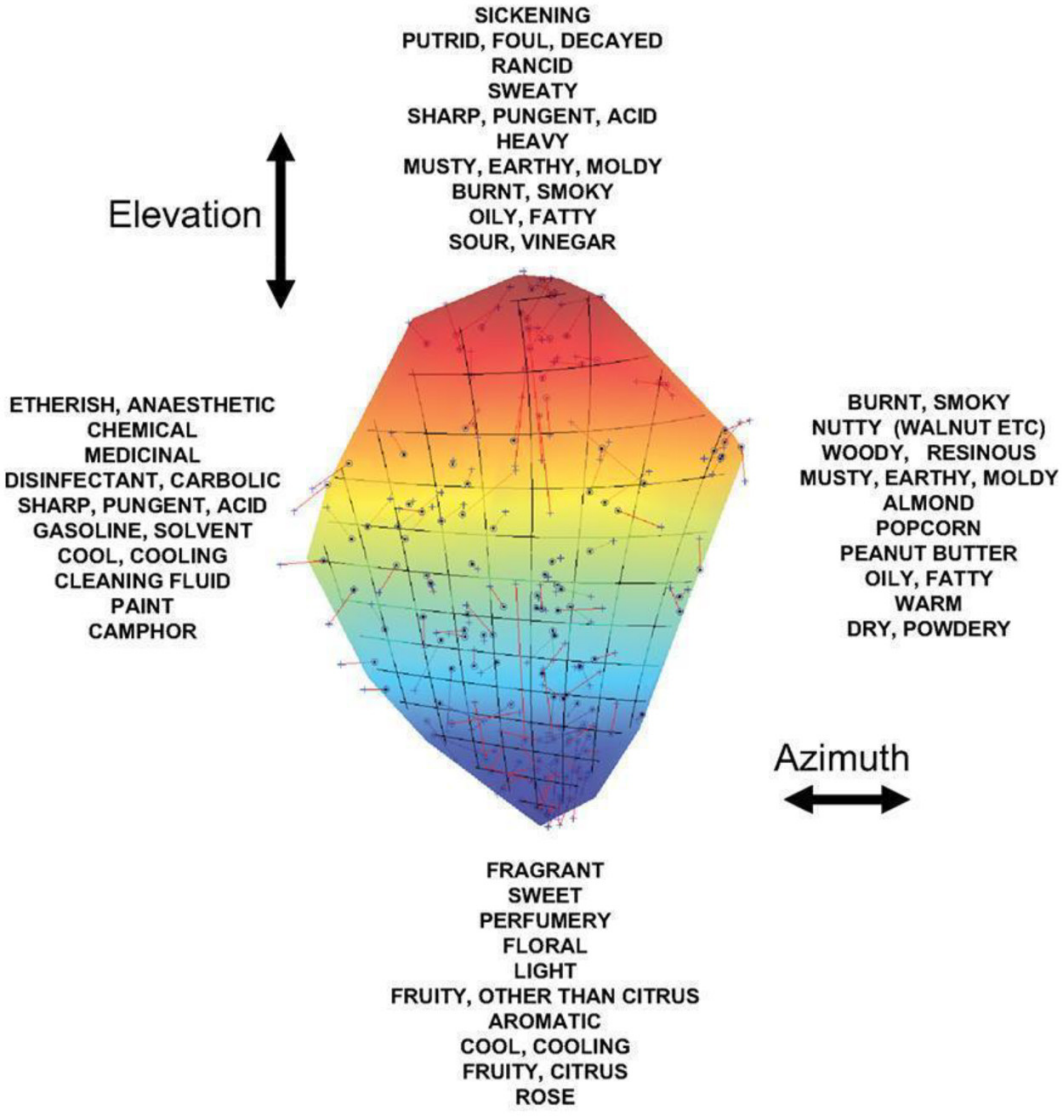
An example that illustrates the potential complexity inherent in the structure of human olfactory space. [From Koulakov et al. (2011)].

Uncertainty concerning the definition, quantity, or quality of dimensions of olfactory experience may potentially hinder the clear establishment of correspondences with well-defined auditory dimensions. Of the very large number of ingredients that are used in professional perfumery, to date only a small number have actually been linked to specific musical parameters. What is more, it is unclear how many of those parameters (i.e., dimensions of olfactory experience) make sense (or are easily interpretable) cross-culturally. That said, it would seem likely that the musical match for the aroma of a red fruit could be generalized from one red fruit to another (although we are not aware of any published evidence on this point). The key point to note here is that for those wanting to design soundscapes to match particular fragrances, there is a need to simplify the space of olfactory descriptors to help make meaningful matches based on the evidence that has already been collected. The dimensionality of olfactory stimuli has long been debated in the philosophy and psychology of perception (e.g., Le Guérer, 2002). While humans can undoubtedly recognize a wide range of different olfactory stimuli, they often struggle to describe them using a consensual set of perceptual dimensions or descriptors. (Note that this difficulty might, in part, reflect a (Western) linguistic/cultural bias (e.g., see Majid & Burenhult, 2014).) Instead, people tend to rely on idiosyncratic strategies, such as referencing odour sources or using gustatory terms (or other sensory features) metaphorically (cf. Uchida et al., 2021). The complexity of olfactory spaces is highlighted in studies like that of Koulakov et al. (2011), which revealed the intricate representations required to map the multidimensional nature of odours (see **Figure 6**).

Currently, there is no universally accepted system for the classification of odours (Auffarth, 2013). Various studies using different data, statistical methods, and descriptive approaches suggest that odours can be grouped in terms of their perceptual similarity (e.g., Civille & Lawless, 1986; Zarzo & Stanton, 2009), and some categories appear reasonably consistent across cultural boundaries (Chrea et al., 2005). These categories include smoky, camphoraceous, fruity, herbaceous, resinous, earthy, and sweet. The concept of ‘primary’ or ‘basic’ odours – similar to primary colours from which all others are derived – has been proposed (e.g., Weiss et al., 2012), but it has failed to establish any widespread acceptance amongst researchers. However, whether compositionality alone is sufficient to define basic odours remains speculative, and unfortunately the idea lacks a clear, testable framework currently.

### Cross-Cultural Factors

There are also some important cross-cultural differences in the naming of odours that should be mentioned. Consider here only how ‘plum’ and ‘dark cherries’, two descriptors that are often found in English wine descriptions, are replaced with ‘Dried Chinese red dates’ and ‘Chinese black tea leaves’ in Chinese (Lockshin et al., 2017). In fact, Jin et al. (2022) conducted a study reporting that 20 of the 54 descriptors from *Le Nez du Vin* (an aroma kit used for wine taster training in olfactory perception and aroma description) were unfamiliar to Chinese participants. The results from the study on *Le Nez du Vin* therefore provide further evidence concerning how cultural differences influence the description of chemosensory experiences (see also Doty et al., 1996). Similar differences are likely to occur when olfactory descriptors are compared across other pairs of languages. Given that Crisinel and Spence (2012) used a subset of *Le Nez du Vin* odorants when establishing musical–olfactory crossmodal correspondences, it might be interesting to conduct a cross-cultural follow-up study to determine whether the same crossmodal matches are observed even when the name commonly associated with a given odour differs by language/culture. Here, it is also relevant to note that in terms of audition and more specifically music, there is limited research investigating individual differences in the crossmodal correspondences with specific auditory stimuli (see Walker, 1997).

### Primary Olfactory Dimensions

**Figure 7.**
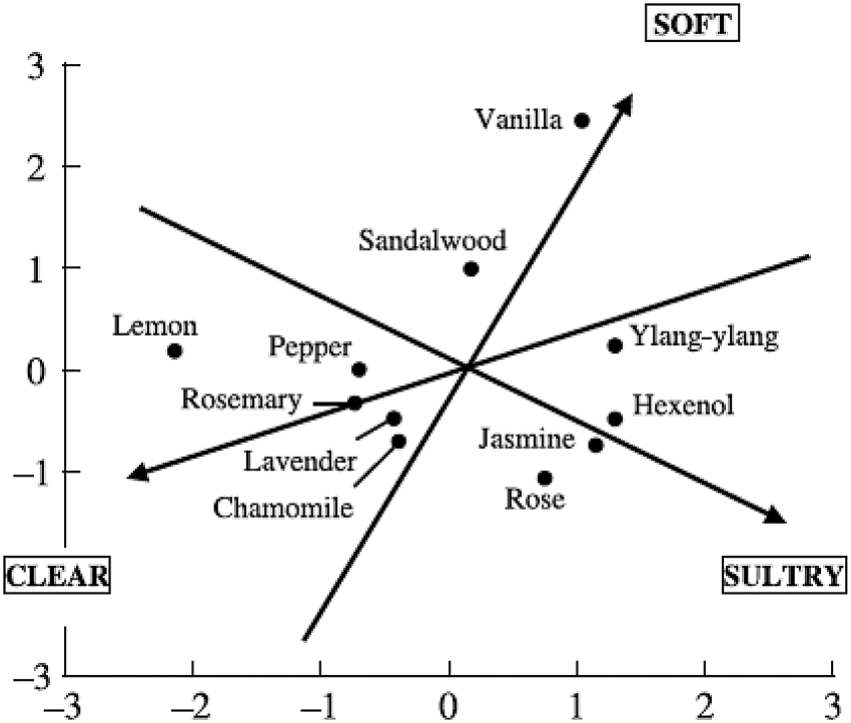
A two-dimensional spatial location of 11 fragrances generated by MDS analysis for an adjective rating scale reported by Higuchi, Shoji, and Hatayama (2002) are shown. MDS, multidimensional scaling.

**Figure 8.**
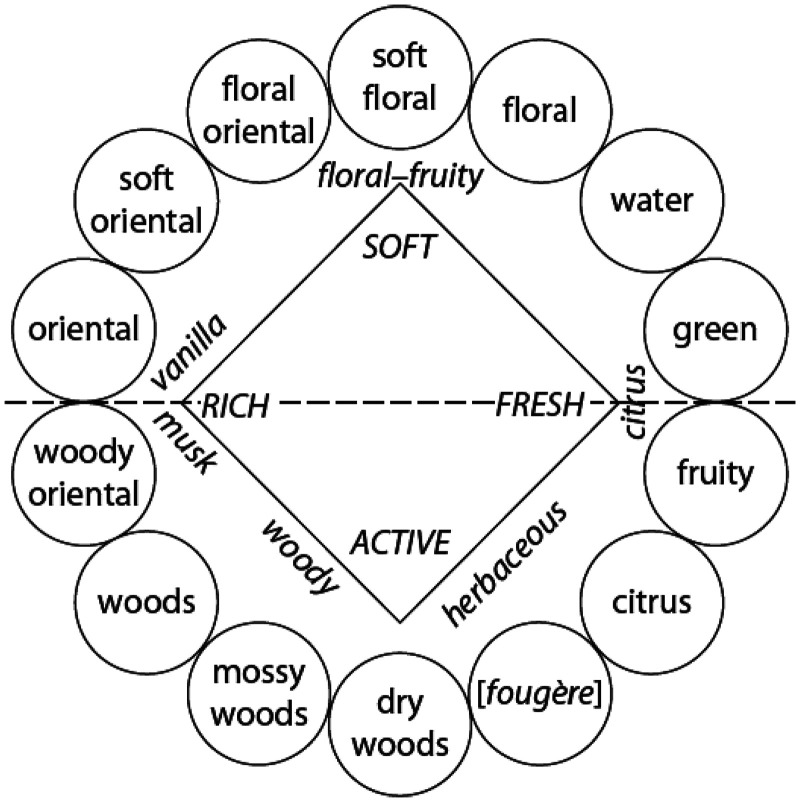
Fragrance wheel from Zarzo and Stanton (2009).

**Figure 9.**
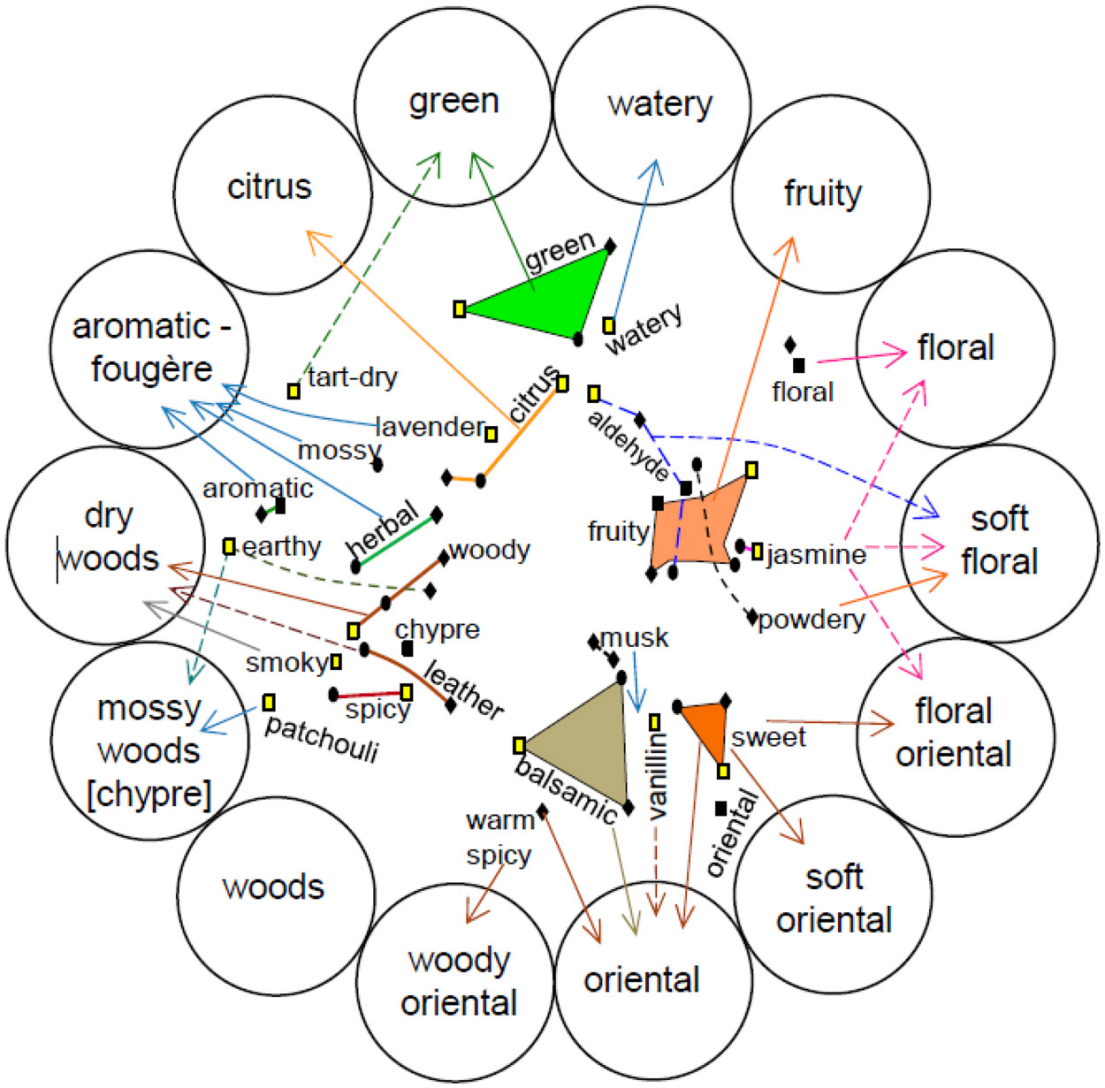
The Fragrance wheel (perfume categories displayed within circles). Arrows indicate the most typical notes of each olfactory class. [Figure reprinted from Zarzo (2020)].

According to Zarzo (2008), who conducted a PCA of 94 commercial fragrances, freshness came out as a primary dimension, followed by whether the cosmetic fragrance was masculine or feminine.^2^ Zarzo (2020) subsequently came up with an alternative approach based on a PCA analysis of 176 commercial fragrances. Zarzo's analysis distinguishes masculine/feminine and day/night-time fragrances. He also suggests a modified fragrance wheel (see **Figures 7** & 8), though it remains unclear whether the regular consumer can necessarily distinguish between all of the descriptors that are presented in the circles, or whether instead a reduced range of e.g., 7 descriptors/categories might be more suitable. As such, one might consider these as two of the key attributes to be evaluated in the Temporal Dominance of Sensations (TDS) analysis of any fine fragrance. ‘Floral’ vs. ‘not floral’ and ‘cool’ vs. ‘warm’ came out as two meaningful axes based on a study of 94 commercial fragrances by Jellinek (1992). Note that green and citrus notes are perceived as fresh and they tend to evaporate quickly, consistent with the light and fresh synonym usage (Mesz et al., 2023). By contrast, odours most dissimilar to fresh are described as complex and full (Zacharakis, 2025). There might also be a link here to sonic matches to ‘heavy’ vs. ‘light’ (Murari et al., 2014), considered similar to the polarity of ‘fresh’ vs. ‘warm’ (see also **Figure 9** for an alternative solution).

## Temporal Aspects of Fragrance and Music Perception

The literature that has been reviewed thus far has mostly only considered appropriate crossmodal matches between sound and scent in the moment. However, the consumer's experience of a fine fragrance, as is the case for music, is likely to evolve over time (Bowman et al., 2012). Indeed, the experience of a fine fragrance is inherently perceived as a temporally evolving event, just as music evolves over time.^3^ For example, top, middle, and bass notes may be integrated in a unitary olfactory Gestalt, just as the various instruments playing in the orchestra may be integrated into a perceptual whole. However, top notes may soon drop out, but it is unclear how that is experienced perceptually (i.e., whether there is some form of olfactory filling-in). In the flavour domain, it is clear that the presence of a volatile compound, by itself, does not guarantee that the consumer will necessarily perceive it (Davidson et al., 1997). When considering the olfactory pyramid (that is often used in perfumery), with top notes (first impressions and volatile notes at the top of the pyramid), so-called heart notes (development and character notes, in the mid-section), and base notes at the bottom of the pyramid (these are the foundation and lasting notes), it remains unclear whether the individual olfactory notes are describing what the base volatiles in the mix are, or whether they are intended to refer to the consumer's perceptual experience of the fragrance, or both. Generally speaking, higher partials have less energy and perceived duration, as is the case for the top notes of a perfume. The challenge is that it is currently simply not possible to predict how a combination of odours will be perceived: That is, will the individual notes remain perceptible and distinctive, or will they merge to deliver a different perceptual experience. Think here only of how distinct notes of citrus, vanilla, and cinnamon when blended may be perceived instead as smelling like cola (what perhaps might be described as a kind of olfactory Gestalt).

The discussion of how multiple olfactory components interact to give rise to a unified percept resonates with the field of auditory scene analysis (Bregman, 1990). The latter field of research explores how the brain interprets complex auditory environments and offers principles for understanding how sound events are perceptually grouped or separated over time. The fusion of sound components begins at a fundamental level – for instance when harmonically related partials with closely aligned temporal envelopes are integrated into the perception of a single tone. This fusion can extend to more complex auditory events, such as chords, formed by the superposition of multiple notes (e.g., C, E, G, Bb, C), or to augmented and even emergent timbres resulting from ingenious orchestration techniques (Sandell, 1995). Perhaps unsurprisingly, the degree of perceived blend depends on musical training, amongst other acoustic factors (McAdams et al., 2025). A further analogy can be drawn between the concept of top, middle, and base notes in perfumery and the frequency spectrum present in musical sounds. Indeed, higher frequencies, at least in naturally produced musical tones, tend to decrease in amplitude and are absorbed more rapidly during transmission through air. At the same time, however, the human auditory system is more sensitive to higher frequencies (with a maximum sensitivity between 3–4 kHz). As such, the presence of high-frequency components within a tone can manifest perceptually even at lower energies. Possibly, humans are likewise more sensitive to top aromatic notes, in which case this would represent an additional analogue to their characteristically faster perceptual fade-out. Elsewhere it has been shown that ethyl isobutyrate (which smells of strawberry) when combined with ethyl maltol (which smells of caramel) gives rise to the perception of pineapple in humans as well as in several other species (Coureaud et al., 2022).

### Future Research Directions

**Figure 10.**
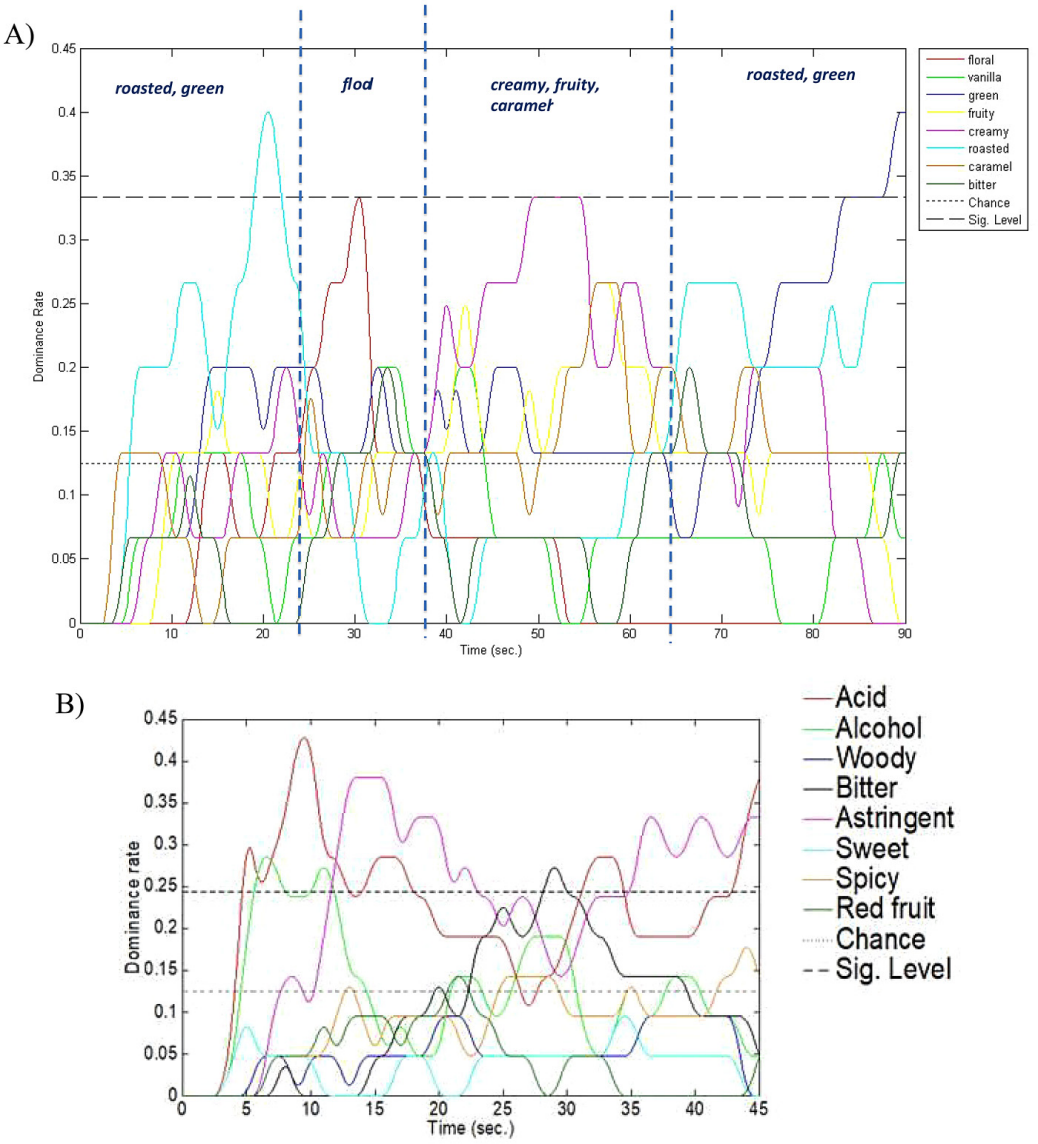
(A) Results of a TDS analysis of the key sensory descriptors associated with a chocolate sample as it melts on the tongue for 90 seconds. [Data taken from an unpublished study with Godiva; see https://www.youtube.com/watch?v = RUbCmyk5EjI&pp = ygUhZ29kaXZhIDcwIGRhcmsgY2hvY29sYXRlIHN5bXBob255; https://www.youtube.com/watch?v = hTgMW7ylSYcv]; (B) Results of a TDS analysis of the key sensory descriptors associated with a mouthful of red wine held in the mouth for 45 seconds. TDS analysis of Manos Negras Pinot Noir 2014 held in the mouth for 45 seconds [Wang, Mesz, Riera, Trevisan, Sigman, Guha, & Spence (2019).] TDS, Temporal Dominance of Sensations.

One way to uncover additional relevant information concerning the temporal evolution of a fragrance would be to conduct a TDS study. The results of such a study could then be used to provide guidelines for the matching soundscape (or musical composition). This approach is one that has been used previously to provide musical recommendations for the sound designer wanting to capture the chemosensory experience of a chocolate as it melts in the mouth over 90 s (see **Figure 10A**), and for wine as it is held in the mouth for 45 s (see **Figure 10B**; see Wang et al., 2019). Interestingly though, as far as we are aware, no one has yet published a study using the TDS approach to investigate the consumer's evolving olfactory perception of a fragrance (Galmarini et al., 2021; see Di Monaco et al., 2014, for a review). As an alternative to conducting a TDS analysis, one might consider whether it is possible to get an approximate idea of the likely temporal evolution of a fragrance from considering the top, middle, and base notes that go into making up the olfactory pyramid for a given fragrance. On the other hand, one might worry that fragrances perhaps do not exhibit the same complex time-varying evolution that one finds in a complex food product (that may be comprised of more than 1,000 distinct volatile compounds; see Spence & Wang, 2018).

In terms of the olfactory descriptors that should be used for the TDS analysis, rather than the specific olfactory notes (e.g., perhaps those taken from the olfactory pyramid associated with a given fragrance), of which there are a huge number of possibilities, and which regular customers might not be able to easily identify, it would presumably make sense to use the key odour descriptor categories that are commonly used in perfumery. Here, though, one immediately runs into a challenge, given that there is little consensus in terms of how olfactory stimuli should be categorized (e.g., see Zarzo, 2008, 2013, 2020; Zarzo & Stanton, 2009). Nevertheless, aromatic, fresh, spicy, and woody would likely be amongst the olfactory category labels in the case of perfumery (see Zarzo, 2008). In particular, Zarzo (2008) highlights the following descriptors: Lavender; Medicinal; Animal; Erogenic; Sweet; Aromatic; Spicy; Dusty; Smoky; Metallic; Citrusy; Minty; Powdery; Floral; Fruity, based on the findings of Boelens and Haring (1981) and Moncrieff (1966).

It would be interesting to consider whether certain fragrances might be associated with a particular musical beat? Are there, for example, fast/slow fragrances, or else waltz- or march-like scents? Relevant here, mean note duration, which may be correlated to tempo in general, was not found to be significantly correlated with olfactory dimensions in Mesz et al.'s (2023) study. Nevertheless, the arousing effect of perfumes should probably relate to musical velocity/tempo. Of course, if the perceived features of a fragrance were to dynamically evolve over time, the matching auditory features/dimensions/stimulus might need to change as well. For example, a composer could use a TDS graph's shifting dominant notes as a guide for creating multi-layered electroacoustic textural compositions or for orchestrating existing pieces with timbres that match the evolving olfactory categories. One other analogue between sound and perfume that could be suggested here concerns the fact that a fragrance, when applied to the skin, evolves differently depending on the individual's skin. As such, the interaction between skin and fragrance could be likened to the interaction between sound and space, where the acoustic characteristics of an environment (e.g., reverberation time, spectral colouration, etc.) significantly influence the final sound that reaches the ear. Once again, this likely adds an additional layer of complexity for those wanting to design soundscapes to match particular fragrances (as experienced on the skin of the wearer).

## Final Considerations

There are a few final issues that should be considered by those wanting to design soundscapes to match specific fragrances. One issue concerns the extent to which the consumer is made aware of the scientific backstory of the project, when attempting to systematically match music to fragrance. The other consideration relates to possible cross-cultural differences in crossmodal correspondences between fragrances (including their scent, but also their other sensory and emotional associations), and music.

Even though the scientific study of crossmodal correspondences can likely help those wanting to design soundscapes to match fragrances, it may be advisable to keep the science aspect in the background, at least if the results of a study of sonic seasoning for chocolate are anything to go by. In particular, a few years ago a study was conducted in one of Dominique Persoone's chocolate shops in Belgium (www.thechocolateline.be). All of the participants who took part in the study tasted the same type of chocolate while listening to the same piece of Brazilian music (a fragment of ‘Vem Morena, Vem’, a song composed by the Brazilian artist Jorge Ben). However, telling the customers that the music was the inspiration for the chocolate led people to enjoy their multisensory tasting experience significantly more than saying that science had proved that the match was a good one (see Reinoso Carvalho et al., 2015). In other words, while science can undoubtedly help with the crossmodal matching process, it may be that consumers resonate more with the artistic inspiration angle (see also Reinoso Carvalho et al., 2016, for a similar example with beer).

Second, the question of cross-cultural similarities and differences is also relevant. Here, it is important to note that while certain crossmodal correspondences appear to be universal (Bremner et al., 2013), there are others, such as, for example, the crossmodal correspondence between colour and flavour which may show at least some degree of cultural variation (e.g., see Levitan et al., 2014; Raevskiy et al., 2022; Shankar et al., 2010). Especially relevant to the theme of the present narrative historical review are any cultural differences in crossmodal correspondences that may exist between music and fragrance. While there is little research that has addressed this question directly, what evidence there is would appear to suggest a high degree of cross-cultural concordance in crossmodal correspondences involving musical inputs (cf. Di Stefano et al., 2024; Di Stefano et al., 2025). So, for example, in one study, both North American and Indian participants were shown to be able to decode the taste (i.e., bitter, sweet, salty, sour) intended by the composer with roughly the same degree of accuracy (Knoeferle et al., 2015). However, as yet, an equivalent cross-cultural study of olfactory-auditory crossmodal correspondences has yet to be published.

One way to think about such cross-cultural similarities and differences is that differences may be more likely to be observed when the correspondence itself is based on semantic knowledge, e.g., of the colour that is normally associated with a specific product, say, whereas cross-cultural generalizability would be expected in the case of those crossmodal correspondences that are based on more abstract stimulus properties. That being said, given that the emotional response to fragrance can differ markedly as a function of culture (see Ayabe-Kanamura et al., 1998a; Ayabe-Kanamura et al., 1998b), then any crossmodal correspondences based on emotional mediation (e.g., Wang & Spence, 2017b) might also be expected to show some degree of predictable cross-cultural difference as well. Note also that the meaning of specific semantic descriptors, such as ‘fresh’, has also been reported to differ as a function of the language spoken (e.g., Zarzo, 2013, 2020). At the same time, however, there are also certain smells such as the odour of vanilla (or vanillin) that appear to be fairly universally liked (Arshamian et al., 2022; Spence, 2022a). Cross-cultural similarities and differences have also been reported in the crossmodal correspondences documented with musical stimuli (e.g., see Walker, 1987).

Another salient consideration is the fact that sound, when negatively perceived, understood as incongruent, or even framed as noise by the brain, could end-up giving rise to undesirable effects. If one thinks of unwelcoming sound as something that distracts or even irritates (when compared to a pleasant sound), it could be hypothesized that such sound would negatively affect cognitive processes, such as attention. For example, in a study by Bravo-Moncayo et al. (2020), passive versus active commercial headphone noise control techniques were applied to a coffee consumption situation. Each participant tasted and rated the same coffee twice while exposed to a loud versus a quieter version of the same background sound of a food court in busy hours. The results revealed that most participants were significantly less sensitive to specific sensory and hedonic attributes of the coffee, including flavour and aroma intensity, under conditions of louder background noise (cf. Spence, 2014, for a review).

One final consideration that is worth making before closing: Given the phenomenon of ‘sensation transference’, the likelihood is that the sonic compositions should be pleasant to listen to, since the emotional associations that the consumer has with soundscape will likely carry over to influence their perception of the fragrance (Reinoso Carvalho et al., 2017; Seo & Hummel, 2011; Velasco et al., 2014; Zhou & Yamanaka, 2018). Put simply, the more the customer likes the music/soundscape, the more they will likely appreciate the fragrance that it is paired with. This is one of the areas where artificial intelligence (AI)-generated soundscapes are likely to struggle. Looking to the future, it may soon be possible to create music (or at least soundscapes) to match a scent, or vice versa, using AI (cf. Murari et al., 2020; Spanio et al., 2025; see also Ward et al., 2022). If so, one direction could be to assess the correspondence between isolated ingredients/aromas (e.g., lavender, tobacco…) that are present in fragrances and acoustic features (e.g., pitch, loudness). Not so far away in the future, it may one day be possible to digitize the sense of smell (though see Spence et al., 2017), and this is where such combinations of features may soon be automatized, and where crossmodal research may increasingly gain interest from different researchers in fields related to immersive digital experiences.

## Conclusions

Based on the evidence that has been reviewed here, it is possible to recommend various musical parameters to match specific elements that constitute commercial fragrances. At the same time, however, this narrative historical review has also highlighted several important challenges that emerge as soon as one tries to jump from the crossmodal correspondences that have been established between individual musical notes and sonic parameters to doing the same thing in the case of a typical commercial fragrance. For one thing, the dimensions of pleasantness and intensity are unlikely to contribute much since these dimensions will likely be approximately matched (and any differences in pleasantness that are observed are likely to reflect individual differences) rather than more basic sensory or higher order consensual crossmodal correspondences. What is more, multi-element commercial fragrances typically contain top, middle, and base notes, again potentially making it somewhat harder to pick a specific sensory match in the auditory domain that will be uniquely felt as corresponding to a given fragrance. Another issue is that odorants tend to mix perceptually in ways that are unpredictable, meaning that it is unclear whether one can necessarily go directly from an olfactory note-musical sound correspondence to assuming that if a perfume contains that note, the crossmodal correspondence will necessarily be preserved in the olfactory mix (or fine fragrance). Nevertheless, despite these challenges, there have been a number of both commercial and more academic attempts to make music that meaningfully corresponds with a given commercial fragrance (or range of fragrances).

Complicating matters further, commercial fine fragrances typically also have a distinctive visual appearance (of the perfume itself and/or its packaging). As yet, it is unclear how these visual aspects may influence (or bias) any olfactory-musical matching. Finally, it is unclear whether the most appropriate level for any crossmodal correspondence is between the sensory attributes of the component stimuli (i.e., fragrance and music) or whether instead the correspondences operate at more of an emotional level (and, in the latter case, whether the emotions that consumers intuitively associate with the fragrance or those associated with the advertising copy / positioning of the fragrance brand).

In contrast to the case of sonic seasoning, where there is widespread agreement on what the basic categories are (i.e., sweet, sour, bitter, salty, umami, and possibly also spicy, creamy, metallic) there is no agreement when it comes to the basic categories that are most appropriate when describing fragrances. What is more, the categories that have been proposed to date would appear to be quite specific to the particular category of product or aromas that happen to be selected. If one restricts oneself specifically to the fragrance category then the sound designer interested in creating a soundscape to match a specific fragrance will need to find the right balance between the number of categories, or dimensions – too few and there is not enough differentiation to work with, too many categories and the assessment procedure may become overly arduous. Moreover, by using meaningful olfactory categories (such as fruity (sweet), fruity (sour), spicy, refreshing, etc.) it may be possible to generalize from exemplar correspondences that have already been documented to others that have not. So, for example, knowing that raspberry, as a fruity aroma, is associated with high pitch and piano notes, means that one can probably assume that pomegranate and blackberry aromas would share a reasonably similar crossmodal mapping even without necessarily needing to test these mappings explicitly.

## Notes

1. International Flavors & Fragrances (https://www.iff.com/) – one of the world's largest fragrance providers.

2. Note that the musical qualities associated with masculine and feminine fragrances were mentioned earlier.

3. There is also a sense in which we are sensitive to subtle differences in the time of arrival of olfactory stimuli during a sniff (Wu et al., 2024).
